# Risk factors for fluoropyrimidine-induced cardiotoxicity in colorectal cancer: A retrospective cohort study and establishment of a prediction nomogram for 5-FU induced cardiotoxicity

**DOI:** 10.3389/fonc.2023.1017237

**Published:** 2023-03-01

**Authors:** Yan Wang, Wenling Wang, Hongming Dong, Gang Wang, Wanghua Chen, Juan Chen, Weiwei Chen

**Affiliations:** ^1^ Department of Oncology, Affiliated Hospital of Guizhou Medical University, Guiyang, China; ^2^ Department of Clinical Medicine, Guizhou Medical University, Guiyang, China; ^3^ Department of Abdominal Oncology, Affiliated Cancer Hospital of Guizhou Medical University, Guiyang, China

**Keywords:** fluoropyrimidine-related cardiotoxicity, risk factor, colorectal cancer, nomogram, prediction model

## Abstract

**Background:**

Fluoropyrimidine is an important component of systemic chemotherapy for colorectal cancer (CRC). Fluoropyrimidine-induced cardiotoxicity (FIC) may result in delay and discontinuation of chemotherapy and, in severe cases, can even be life-threatening. To date, risk factors for FIC have not been well identified. This cohort study aimed to identify the predictors of FIC in CRC patients and develop a risk prediction nomogram model.

**Methods:**

Between January 1, 2018 and December 31, 2020, colorectal cancer patients who received 5-fluoropyrimidine(5-Fu)/capecitabine-based chemotherapy in Affiliated Cancer Hospital of Guizhou Medical University were included. FIC was defined as an adverse cardiovascular event related to fluoropyrimidine that occurred during or within four weeks of completing chemotherapy. Risk factors were determined by LASSO algorithm and multivariate logistic regression analysis. Nomogram for predicting 5-Fu-induced cardiotoxicity was established and internally validated. The concordance index (C-index) and calibration curve were used to evaluate the nomogram’s discrimination and accuracy.

**Results:**

A total of 916 patients were included for analysis, and 200 [21.8%,95% confidence interval (CI):19.12%-24.47%] experienced FIC. LASSO algorithm and multivariate logistic regression analysis determined that chemotherapy ≤3 cycles (OR=4.694, 95%CI=3.184-6.92), age≥ 60 (OR=1.678, 95%CI=1.143-2.464), BMI>22.97 (OR=1.77, 95%CI=1.202-2.606), and simultaneous use of bevacizumab (OR=2.922, 95%CI=1.835-4.653) were significant risk factors, and were included in the prediction model for 5-Fu induced cardiotoxicity. The C-index (95%CI) was 0.751 (0.706-0.795) by internal validation. For patients treated with capecitabine-based regimen, the incidence of FIC increased with the absolute value of neutrophils (OR=5.177, 95%CI=1.684-15.549) and eosinophils (OR=3.377,95% CI=1.237-9.22).

**Conclusions:**

Our study identified risk factors for FIC and established a prediction nomogram model based on chemotherapy cycle, age, BMI and use of target therapy for 5-FU induced Cardiotoxicity. The discriminative prediction model can be used for patient counselling and risk-stratification before undergoing chemotherapy in colorectal cancer.

## Background

Globally, colorectal cancer (CRC) is the third most common cancer with the second highest mortality rate (1). Chemotherapy is a critical part of colorectal cancer treatment, significantly reducing the risk of disease recurrence and mortality, and ultimately improving the quality of life (2). Nevertheless, chemotherapeutic agents can cause both reversible and irreversible cardiotoxicity, ultimately increasing the incidence of cardiovascular diseases (2–4). The mortality of cardiovascular diseases currently ranks second in tumor patients, exceeded only by tumor recurrence (5). Fluoropyrimidine remains a cornerstone of chemotherapy for colorectal cancer, which is principally administered as either 5-fluorouracil (5-FU) or its oral prodrug, capecitabine (6). Fluoropyrimidine-induced cardiotoxicity (FIC), the incidence of which is only exceeded by anthracyclines, can affect coronary blood flow or directly damage the myocardium, leading to myocardial infarction, cardiomyopathy, cardiogenic shock or even death (7–10). FIC not only can be life-threatening, but also leads directly to the withdrawal of patients from the optimal chemotherapeutic regimen. Therefore, risk evaluation of FIC is essential for clinical treatment and management. Here, we aimed to identify the incidence and predictors of FIC in a large sample of patients with colorectal cancer and to develop and validate a prediction nomogram model intended to assist clinicians in assessing the risk of FIC in this population.

## Patients and methods

### Selection of patients

The study was approved by the institutional ethics committee of the Affiliated Cancer Hospital of Guizhou Medical University (FZ 2021-11-298) and the institutional ethics committee waived the requirement for informed consent given the deidentified data. The study followed the Strengthening the Reporting of Observational Studies in Epidemiology (STROBE) reporting guideline. Patients who were diagnosed with colorectal adenocarcinoma and received 5-Fu/capecitabine-based chemotherapy at Affiliated Cancer Hospital of Guizhou Medical University from January 1, 2018 to December 31, 2020 were included. Similar to the approach used in prior studies, the inclusion criteria were as follows: (1) pathologically confirmed colon or rectal adenocarcinoma; (2) no severe medical complication prior to treatment (uncontrolled serious high blood pressure, acute stage of myocardial infarction, unstable angina pectoris, uncorrected severe cardiac insufficiency, or serious arrhythmia); (3) normal blood count, hepatorenal function, and electrocardiogram, and left ventricular ejection fraction (LVEF)≥50%; and (4) received fluoropyrimidine-based chemotherapy (11, 12).

### Variables

Collected data included demographic factors, medical complications prior to treatment, laboratory tests within 3 days before treatment, smoking and alcohol history, treatment regimen and cycle, and Cancer staging based on the 8th edition of the American Joint Committee on Cancer (AJCC)/TNM system (13). The medical complications we focused on were recorded in electronic medical record system, mainly including: history of cardiovascular disease (acute myocardial infarction, ischemic heart disease, high blood pressure, cardiac failure or stroke), history of endocrinopathy (diabetes, hyperlipidemia, hyperthyroidism and hypothyroidism); in our study, it is represented by yes or no; Results of laboratory tests recorded in electronic medical record system, containing blood count, hepatorenal function, and electrocardiogram, and left ventricular ejection were obtained.

FIC was defined as significant symptoms of likely cardiac origin (chest pain, palpitations, dyspnoea, or cardiogenic shock), electrocardiographic abnormalities (ST-T changes or arrhythmia), abnormal echocardiogram, or obvious elevation of myocardial enzymes that developed during treatment (14–18). These changes occurred during 5-FU/capecitabine treatment or within 4 weeks following completion of treatment but were not present before treatment. Cases of suspected FIC were further reviewed by cardiologists who rendered the final decision of whether FIC was diagnosed. FIC was graded according to Common Terminology Criteria for Adverse Events (CTCAE) (19) for both 5-FU and capecitabine groups.

### Statistical analysis

Categorical data were presented as numbers and percentages. For continuous variables, the value of normal distribution was expressed as mean± SD, and the value of non-normal distribution as medians and interquartile ranges (IQRs). The χ2 test or Fisher’s exact tests were used for univariate analysis. The least absolute shrinkage and selection operator (LASSO) regression method was employed for predictor selection. Significant variables in the LASSO analysis were subjected to multivariate logistic regression analysis by backward stepwise selection under the Akaike information criterion (AIC) (20). Receiver operating characteristic (ROC) curve was used to determine the cut-off value of continuous variables. Continuous variables were divided into high or low groups according to the optimal cut-off value. Variables that were statistically significant in the multivariate logistic regression analysis were determined as independent risk factors of the FIC. Nomogram was constructed according to the logistic regression analysis. The concordance index (C-index) applied to evaluate the discriminatory ability of the nomogram, was used to evaluate discriminative ability. C-index values greater than 0.7 suggested a reasonable estimation (19). The calibration curve was used to evaluate the nomogram’s accuracy. Statistical analyses were performed using R software (version 3.6.3; https://www.r-project.org). A value of P <0.05 was considered statistically significant.

## Results

### Patient characteristics

**Figure 1:**
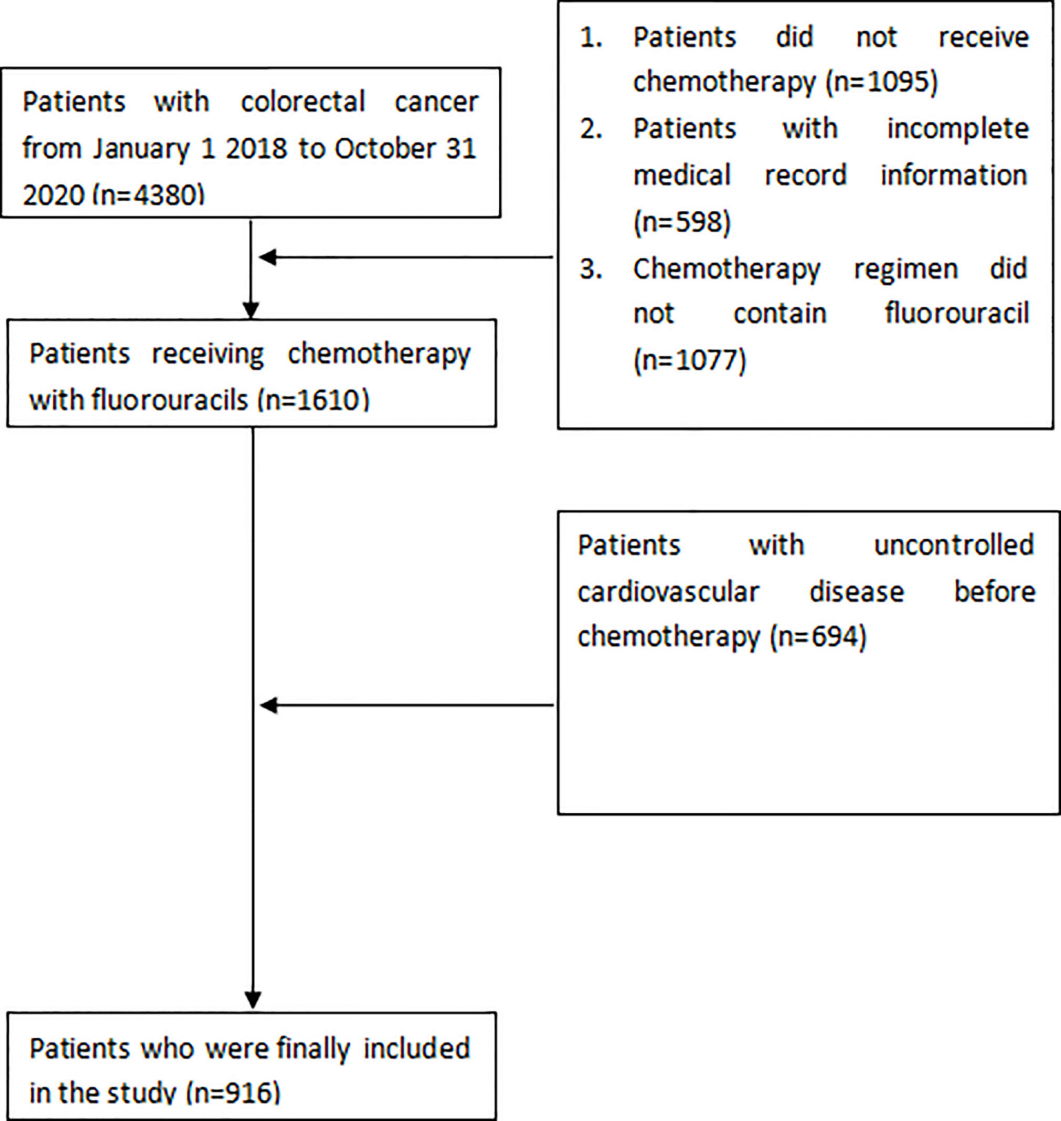
Flow chart of enrolment.

We reviewed 4380 patients diagnosed as colorectal cancer in Affiliated Cancer Hospital of Guizhou Medical University between January 1, 2018, and December 31, 2020. **Figure 1** shows the flow chart for enrolment. The study finally included 916 patients, of whom 754 (82.3%) received the 5-FU-based chemotherapy regimen, and 162 (17.7%) received capecitabine-based chemotherapy. Patients’ characteristics prior to treatment are shown in **Table 1**.

**Table 1:** Patients’ characteristics.

Characteristics	5-FU patients n = 754 (%)	Capecitabine patients n = 162 (%)
Sex, n (%)		
male	469 (62)	107 (66)
female	285 (38)	55 (34)
Age, Median (Q1, Q3)	56 (48, 64)	59 (50.25, 66)
BMI, Median (Q1, Q3)	21.81 (20.03, 24.22)	22.29 (20.41, 24.09)
Tumor location		
Colon	423 (56)	98 (60)
Rectum	331 (44)	64 (40)
Clinical Stage, n (%)		
I	16 (2)	2 (1)
II	130 (17)	50 (31)
III	372 (49)	83 (51)
IV	236 (31)	27 (17)
SBP, Median (Q1, Q3)	118 (110, 123)	120 (111, 134.5)
DSP, Median (Q1, Q3)	70 (70, 78)	74 (70, 83)
History of Cardiovascular Disease, n (%)		
no	599 (79)	93 (57)
yes	155 (21)	69 (43)
History of Endocrine Disease, n (%)		
no	616 (82)	134 (83)
yes	138 (18)	28 (17)
Alcohol, n (%)		
no	409 (54)	84 (52)
yes	345 (46)	78 (48)
Smoking, n (%)		
no	398 (53)	77 (48)
yes	356 (47)	85 (52)
Chemotherapy Regimen, n (%)		
multidrug	722 (96)	152 (94)
single drug	32 (4)	10 (6)
Targeted Drug, n (%)		
no	595 (79)	157 (97)
Bevacizumab	122 (16)	4 (2)
cetuximab	37 (5)	1 (1)
Chemotherapy Cycle, Median (Q1, Q3)	4 (2, 7)	4 (2, 6)
FIC		
Yes	156 (20.7%)	44 (27.2%)
No	598 (79.3%)	118 (72.8%)
CTCAE, n (%)		
0	598 (79.3)	118 (72.8)
1	46 (6.1)	17 (10.5)
2	108 (14.3)	24 (14.8)
3	0 (0)	2 (1.2)
4	2 (0.3)	1 (0.6)

### Incidence and presentation of FIC

**Figure 2:**
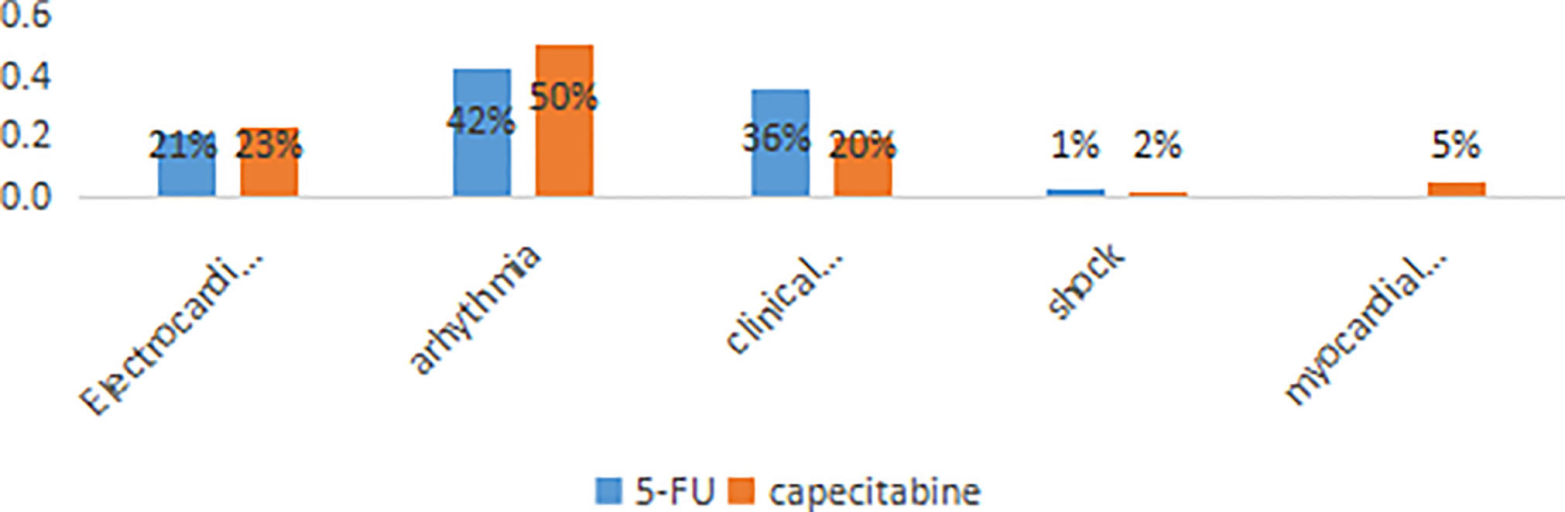
The distribution of the different manifestations of cardiotoxicity. The percentages shown on each bar reflect the distribution of symptoms among the 200 patients with cardiotoxicity.

FIC was diagnosed in 200 patients (21.8%), of whom 156 (78%) occurred in the 5-FU group and 44 (22%) in the capecitabine group. In the 5-FU group, 66/156 (42.3%) experienced arrhythmia (nodal tachycardia, bradycardia, atrioventricular block, premature beat, atrial fibrillation or ventricular fibrillation); 56/156 (35.9%) suffered chest pain and breathing difficulties; 32/156 (20.5%) manifested only ST-T changes (ST-T elevation or depression); and 2/156 (1.3%) suffered myocardial infarction. While in the capecitabine group, 22/44 (50%) had arrhythmia, 10/44 (22.7%) experienced ST-T changes, 9/44 (20.5%) had chest pain and breathing difficulties, 1/44 (2.3%) suffered shock, and 2/44 (4.5%) had myocardial infarction (**Figure 2**).

### Univariate analysis

The results of the univariate logistics analyses are listed in **Table 2**. The variables significantly associated with FIC (p<0.05) in 5-FU group were age, BMI, past history of cardiovascular disease, number of chemotherapy cycles, treatment with targeted drugs, neutrophils(NE), systemic immune-inflammation index(SII), neutrophil to lymphocyte ratio(NLR), creatinine and calcium levels. In the capecitabine group, chemotherapy cycle, white blood cells(WBC), neutrophils(NE), eosinophils(Eos), monocytes(M), NLR, prognostic nutritional index (PNI), SII, Pan-Immune-Inflammation Value(PIV), and neutrophil-to-albumin ratio(NAR) were correlated with FIC (p<0.05).

**Table 2:** Univariate analysis of risk factors.

Variables	5-FU Patients without cardiotoxicity 598 (79.3%)	5-FU Patients with cardiotoxicity 156 (20.7%)	p	Capecitabine Patients without cardiotoxicity 118 (72.8%)	Capecitabine Patients with cardiotoxicity 44 (27.2%)	p
Sex, n (%)			0.648			0.339
male	369 (62)	100 (64)		81 (69)	26 (59)	
Female	229 (38)	56 (36)		37 (31)	18 (41)	
Age, Median (Q1, Q3)	55.5 (47, 63)	61 (51, 67)	< 0.001	59 (50, 66)	59 (51.75, 64)	0.648
BMI, Median (Q1, Q3)	21.64 (19.95, 23.88)	22.82 (20.31, 25.25)	0.013	22.48 (20.76, 24.14)	21.29 (19.98, 23.74)	0.154
SBP, Median (Q1, Q3)	118 (110, 122)	120 (110, 126)	0.188	120 (110, 134.5)	120 (114.5, 132.25)	0.573
DSP, Median (Q1, Q3)	70.5 (70, 78)	70 (68, 78)	0.634	74.5 (69.25, 82.75)	73 (70, 83.25)	0.751
Stage, n (%)			0.223			0.602
I	13 (2)	3 (2)		1 (1)	1 (2)	
II	111 (19)	19 (12)		38 (32)	12 (27)	
III	294 (49)	78 (50)		58 (49)	25 (57)	
IV	180 (30)	56 (36)		21 (18)	6 (14)	
Cardiovascular and Cerebrovascular, n (%)			0.02			0.423
no	486 (81)	113 (72)		65 (55)	28 (64)	
yes	112 (19)	43 (28)		53 (45)	16 (36)	
Endocrine, n (%)			0.651			1
no	491 (82)	125 (80)		98 (83)	36 (82)	
yes	107 (18)	31 (20)		20 (17)	8 (18)	
Alcohol, n (%)			0.983			0.809
no	325 (54)	84 (54)		60 (51)	24 (55)	
yes	273 (46)	72 (46)		58 (49)	20 (45)	
Smoking, n (%)			0.57			1
no	312 (52)	86 (55)		56 (47)	21 (48)	
yes	286 (48)	70 (45)		62 (53)	23 (52)	
Chemotherapy regimens, n (%)			0.402			0.289
multidrug	575 (96)	147 (94)		109 (92)	43 (98)	
single drug	23 (4)	9 (6)		9 (8)	1 (2)	
Targeted Drug, n (%)			< 0.001			0.317
no	494 (83)	101 (65)		115 (97)	42 (95)	
anti-VEGFR	78 (13)	44 (28)		3 (3)	1 (2)	
cetuximab	26 (4)	11 (7)		0 (0)	1 (2)	
Cycle, Median (Q1, Q3)	4 (3, 8)	2 (2, 4)	< 0.001	4(2, 6)	4(2.5, 6)	0.005
NLR, Median (Q1, Q3)	0.13 (0.08, 0.21)	0.15 (0.09, 0.23)	0.02	0.11 (0.08, 0.17)	0.2 (0.12, 0.31)	< 0.001
PLR, Median (Q1, Q3)	8.43 (5.99, 12.16)	9.52 (6.88, 13.15)	0.072	8.57 (6.22, 11.86)	10.95 (7.54, 19.62)	0.007
SII, Median (Q1, Q3)	27.7 (15.64, 52.81)	33.8 (18.91, 61.28)	0.045	26.77 (16.85, 44.32)	48.59 (28.1, 96.74)	< 0.001
PIV, Median (Q1, Q3)	12.02 (6.4, 29.55)	15.46 (7.94, 29.9)	0.069	179.2 ± 46.32	153.91 ± 50.54	0.005
PNI, Mean ± SD	178.89 ± 49.93	167.68 ± 48.08	0.011	11.27 (7.09, 20.68)	16.83 (11.83, 54.21)	0.006
MLR, Median (Q1, Q3)	0.02 (0.01, 0.03)	0.02 (0.01, 0.03)	0.042	0.02 (0.01, 0.02)	0.02 (0.01, 0.03)	0.229
NAR, Median (Q1, Q3)	0.08 (0.06, 0.11)	0.08 (0.07, 0.12)	0.068	0.08 (0.06, 0.1)	0.11 (0.08, 0.14)	< 0.001
WBC, Median (Q1, Q3)	5.62 (4.66, 6.96)	5.96 (4.6, 7.45)	0.21	5.4 (4.51, 6.54)	6.53 (5.53, 7.82)	< 0.001
N, Median (Q1, Q3)	3.33 (2.51, 4.48)	3.61 (2.72, 4.87)	0.065	3.15 (2.46, 4.08)	4.57 (3.39, 5.64)	< 0.001
L, Median (Q1, Q3)	27 (20.62, 33.5)	25.5 (19.35, 31.1)	0.019	27.63 ± 9.14	22.48 ± 9.87	0.004
M., Median (Q1, Q3)	0.46 (0.35, 0.58)	0.46 (0.37, 0.59)	0.426	0.19 (0.12, 0.28)	0.28 (0.2, 0.4)	0.002
EOS., Median (Q1, Q3)	0.2 (0.13, 0.31)	0.2 (0.14, 0.35)	0.133	4.3 (3, 6.15)	5.65 (4.3, 8.12)	0.004
HGB, Median (Q1, Q3)	126 (111, 139)	130 (111.75, 142.25)	0.08	124.5 ± 22.6	123.16 ± 24.43	0.752
P, Median (Q1, Q3)	229 (182, 277.75)	225.5 (187, 277)	0.827	223.5 (183.5, 300)	251.5 (201.5, 290.5)	0.291
ALB, Mean ± SD	41.56 ± 3.91	41.27 ± 4.16	0.424	41.03 ± 4.34	41.51 ± 4.41	0.536
AST, Median (Q1, Q3)	20 (16, 26)	20.39 (16, 27)	0.686	20 (16.01, 27.45)	17.55 (15.78, 25.7)	0.467
ALT, Median (Q1, Q3)	17 (11.9, 26)	18.02 (12, 27)	0.571	17.45 (12, 25.62)	15.7 (12, 25.4)	0.967
LDH, Median (Q1, Q3)	172.08 (147.23, 203)	172 (149.37, 209.29)	0.713	165.25 (145.32, 187.8)	168.5 (148, 192)	0.517
UA, Median (Q1, Q3)	172.08 (147.23, 203)	172 (149.37, 210.04)	0.658	165.25 (145.32, 187.8)	168.5 (148, 192)	0.517
Scr, Median (Q1, Q3)	65 (55.89, 74.94)	67.25 (58, 79.62)	0.023	68.8 (58.02, 79.15)	65.7 (57.6, 78.55)	0.527
BUN.Scr, Median (Q1, Q3)	0.07 (0.06, 0.09)	0.07 (0.06, 0.09)	0.516	0.07 (0.06, 0.09)	0.07 (0.06, 0.08)	0.713
K, Median (Q1, Q3)	4 (3.79, 4.21)	4 (3.71, 4.25)	0.838	3.95 ± 0.44	3.88 ± 0.42	0.373
Ca, Mean ± SD	2.35 ± 0.15	2.33 ± 0.13	0.041	2.31 ± 0.14	2.32 ± 0.14	0.881
GLU, Median (Q1,Q3)	4.71 (4.36, 5.21)	4.82 (4.44, 5.42)	0.077	4.94 (4.55, 5.76)	4.74 (4.33, 5.57)	0.194

NLR =N/L, PLR= P/L, SII = (P × N)/L, PIV= (N ×P × M)/L, PNI=ALB+5 ×P × L, MLR== M/L, NAR= N/ALB.

### Multivariate analysis

**Figure 3:**
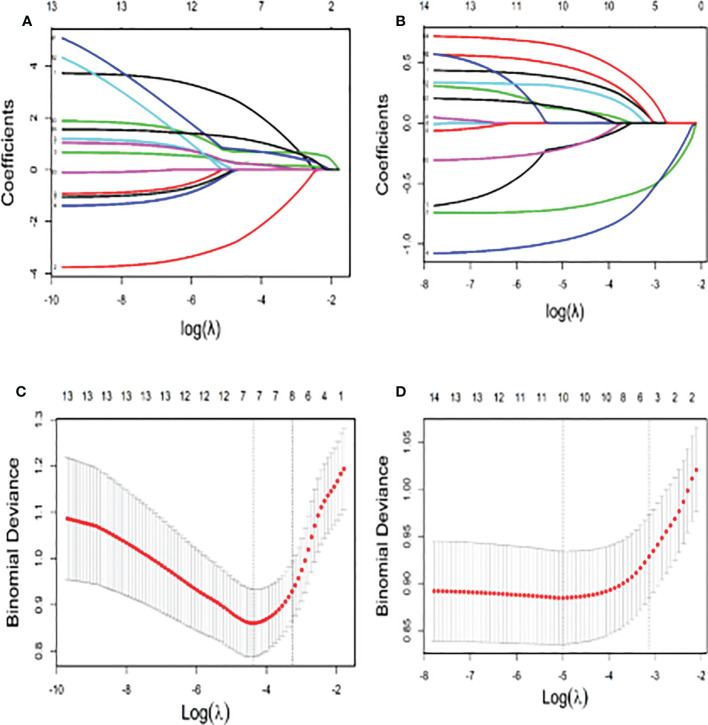
The Lasso Model for Screening of Factors Related to Cardiotoxicity **(A)** The number of variables screened by the 10-fold cross validation in the capecitabin chemotherapy group; **(B)** The number of variables screened by the 10-fold cross validation in the 5-fluorouracil chemotherapy group; **(C)** The coefficient shrink path in the capecitabin chemotherapy group; **(D)** The coefficient shrink path in the capecitabin chemotherapy group).

The significant continuous variables were converted to categorical variables according to the ROC curve. The optimal cut-off points are shown in **Appendix 1**. The LASSO analysis suggested that chemotherapy cycle, WBC, NLR, PIV, NE, and Eos were associated with 5-Fu induced cardiotoxicity. While in the capecitabine group, age, BMI, chemotherapy cycle, and targeted drugs were correlated with capecitabine induced cardiotoxicity (**Figure 3**). Subsequently, all VIFs (Variance Inflation Factor, VIF) were found to be less than ten, which means there existed no collinearity among variables (**Figure 3**).

As shown in **Table 3**, the independent risk factors for FIC identified by multivariate logistic regression analysis in the 5-FU group were: number of chemotherapy cycle ≤ 3 (OR=4.694,95% CI=3.184-6.92, p<0.001), age>60 years old (OR=1.678, 95%CI=1.143-2.464, p=0.008), BMI≥22.97 (OR=1.77, 95%CI=1.202-2.606, p=0.004), and use of bevacizumab (OR=2.922, 95%CI=1.835-4.653, p<0.001. The risk of FIC tended to be higher in patients receiving cetuximab (OR=2.18, 95% CI=0.994-4.793, p=0.051). While in the capecitabine group, the risk of FIC increased by 3.377-fold (OR=3.377, 95%CI=1.237-9.22, p=0.018) and 5.177-fold (OR=5.177,95%CI=1.684-15.549, p=0.004) per unit increase in eosinophils and neutrophils, respectively (**Table 4**).

**Table 3:** Risk factors for FIC by multivariate logistic regression analysis: 5-FU Group.

	B	SE	z	P	OR	95CI%
Age	0.517674597	0.195956726	2.641780188	0.008247156	1.678	1.143-2.464
BMI	0.570926911	0.197438788	2.891665401	0.003832058	1.77	1.202-2.606
Cycle	1.546226972	0.198039806	7.807657468	<0.001	4.694	3.184-6.92
bevacizumab	1.072429536	0.23731897	4.518937255	<0.001	2.922	1.835-4.653
cetuximab	0.780438191	0.40135122	1.944526769	0.051831961	2.182	0.994-4.793

B, regression coefficient; SE, standard error of regression coefficient; OR, odds ratio; CI, confidential interval.

N = 754.

**Table 4:** Risk factors for FIC, multivariate logistic regression: Capecitabine Group.

	B	SE	z	P	OR	(95%CI)
NE	1.632524203	0.567066485	2.878893825	0.003990727	5.117	(1.684-15.549)
EOS	1.217105702	0.512373628	2.375426126	0.017528701	3.377	(1.237-9.22)

B, regression coefficient; SE, standard error of regression coefficient; OR, odds ratio; CI, confidential interval.

N = 162.

### Construction and internal validation of nomograms

**Figure 4:**
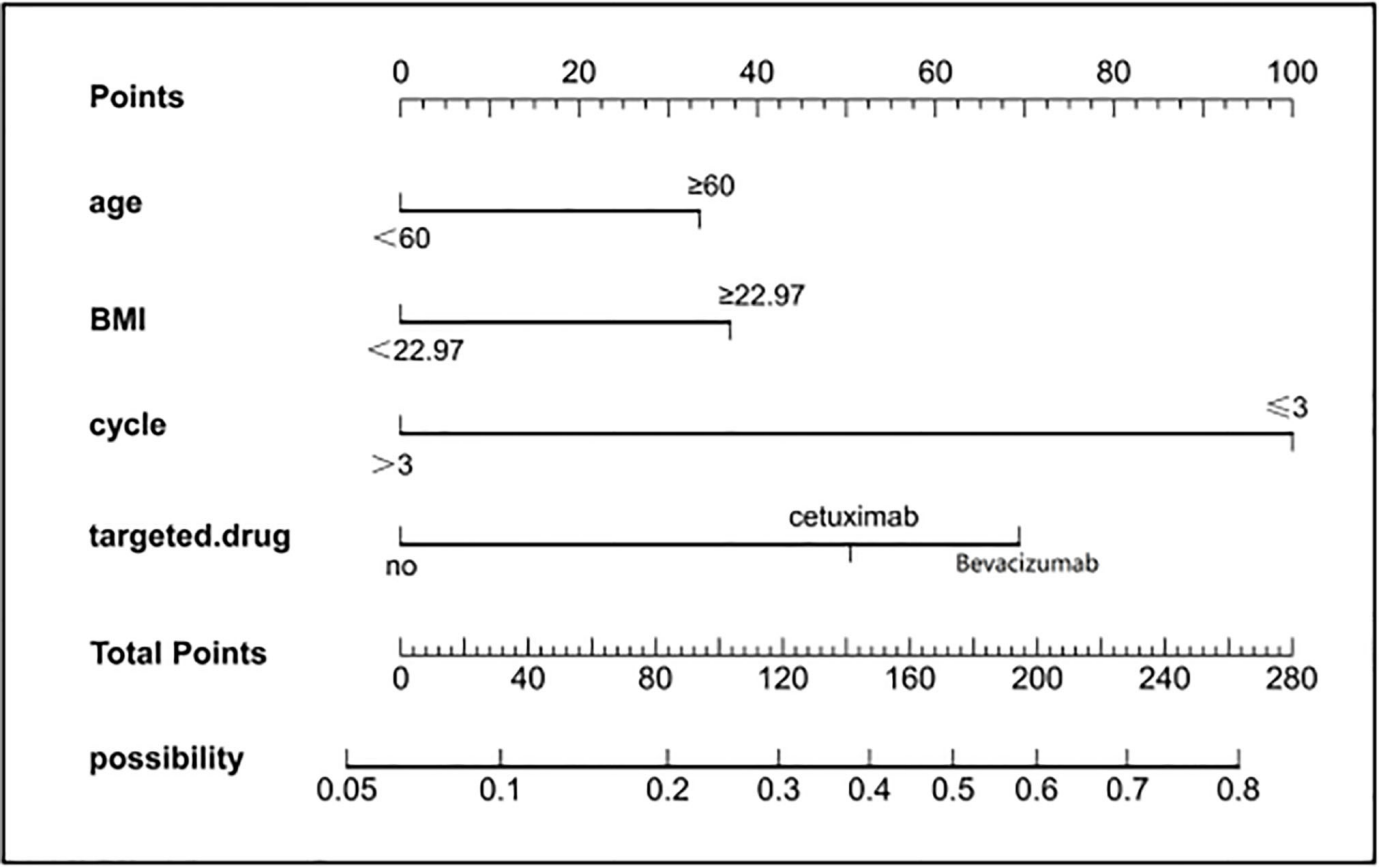
Nomogram for prediction of FIC risk and its predictive performance. First, find the points for each predictor (variable) of a patient on the uppermost rule; then, add all points to calculate the “total points” finally find the corresponding predicted probability of FIC on the lowest rule.

**Figure 5:**
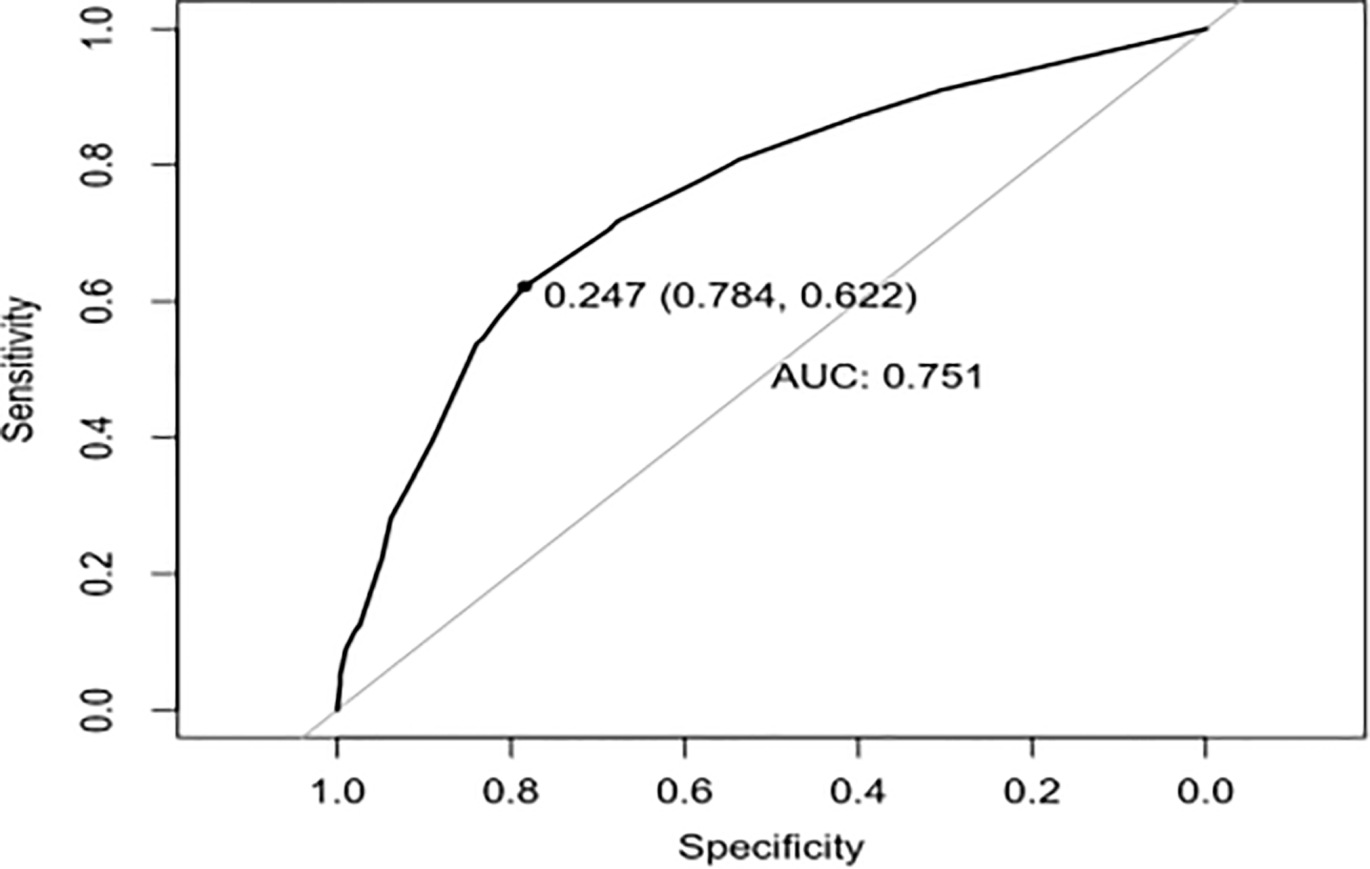
The ROC Curve for the Distinctive Abilities of the Multiple Regression Model.

**Figure 6:**
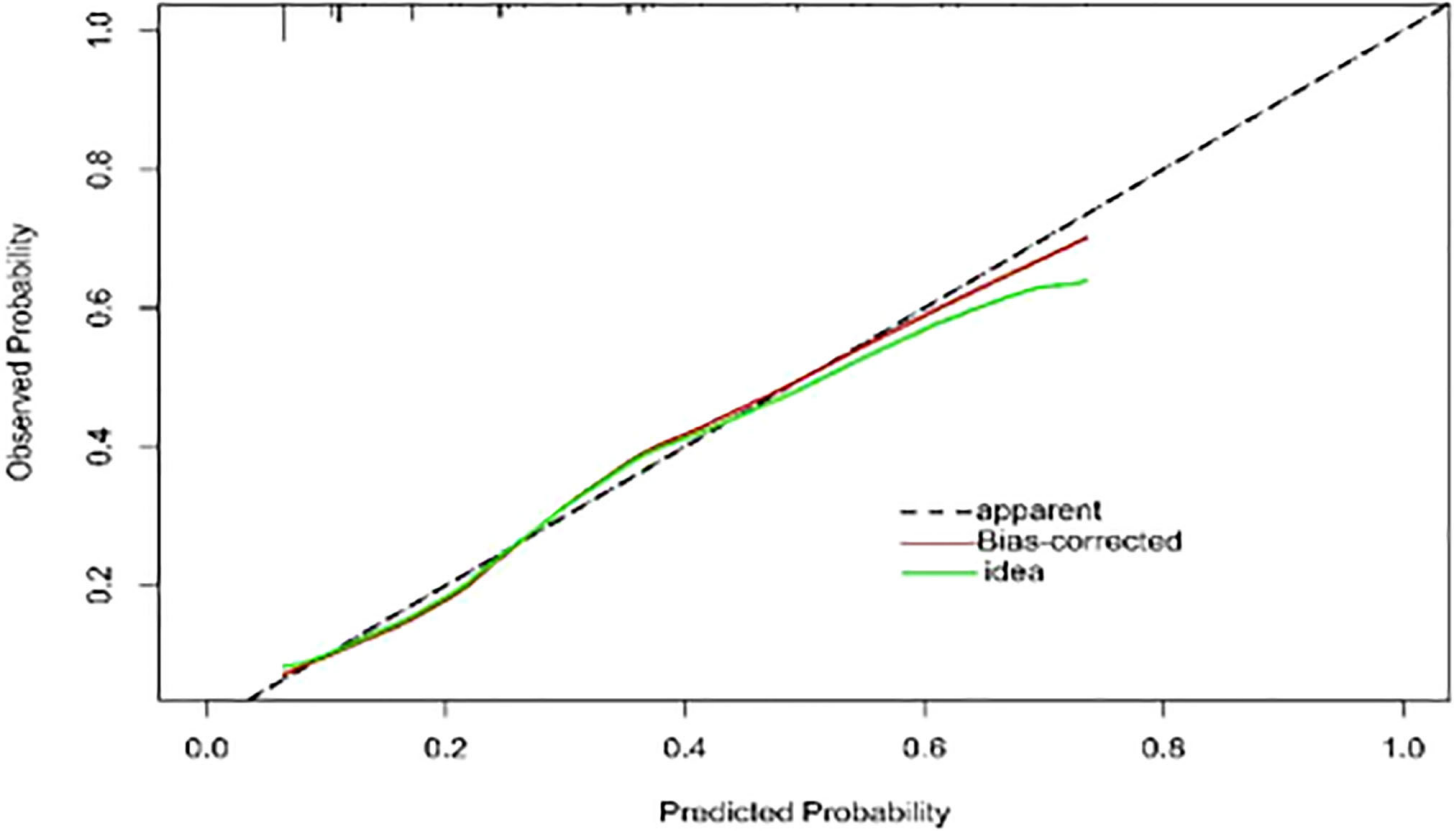
Calibration curve of the model. The calibration of the model in line with the agreement between predicted and observed outcomes of FIC. The Y-axis represents the actual FIC rate. The X-axis represents the predicted risk of FIC. The dotted line represents a perfect prediction by an ideal model. The red line represents the performance of the model, with a closer fit to the shadow line representing a better prediction. FIC, The fluoropyrimidine-induced cardiotoxicity.

To provide physicians with a quantitative tool for individualized prediction of 5-fluorouracil induced cardiotoxicity, a nomogram was constructed according to results of multivariable logistic regression (**Figure 4**). The point score for each parameter was obtained through correlation of each variable to the value of the small ruler (top line of the Nomogram). Each predictive variable had its corresponding score on the points scale. The individual scores corresponding to the independent predictive variables were added to get the total score. The higher the total score, the higher the risk of FIC. Then a perpendicular line was drawn from the total points scale to the probability of FIC on the lowest rule to obtain the probability of cardiotoxicity occurring with an individual patient. The predictive accuracy of this model was then assessed, and the AUC of the nomogram was 0.751. A bootstrap resampling was then used to evaluate the efficacy of the constructed nomogram model, the C-index (95%CI) of the model was 0.751 (0.706-0.795) through the internal validation, which demonstrated the discriminatory ability of this model (**Figure 5**). The calibration curve were used to evaluate the nomogram’s accuracy (**Figure 6**). A Nomogram was not constructed for the capecitabine group as the sample size was deemed insufficient.

## Discussion

In the present retrospective cohort study, we evaluated the high-risk factors for FIC among colorectal cancer patients. We found that in the patients using 5-FU-based chemotherapy, FIC was more likely to occur in the early duration of chemotherapy (chemotherapy cycle ≤3), in patients with age ≥60, BMI≥22.97, or use of targeted drugs. While in the capecitabine group, as the neutrophil and eosinophilic granulocyte counts increased, so did the incidence rate of FIC. Furthermore, we developed a nomogram for predicting FIC in patients using 5-FU-based chemotherapy, which had good discriminative power.

Cardiotoxicity has been one of the common toxic side effects of fluoropyrimidine. In 1969, Gaveau et al. first reported FIC (21), and subsequently there have been an increasing number of similar reports. The reported incidence of FIC related specifically to 5-FU among the European and American varied from 7 to 19.9%, with the oral prodrug capecitabine having an even higher incidence of 34.6% (11, 22–24). According to a retrospective cohort study of 129 Chinese patients with gastric cancer, 38(29.5%) patients developed FIC (25). In our study, the incidence rate of cardiotoxicity was 21.8% (200/916), with 156 (20.6%) in the 5-FU group and 44 (27.1%) in the capecitabine group, suggesting that the incidence of cardiotoxicity in the Chinese population might be higher than that in the European and American populations. The incidence rate of FIC in the capecitabine group in our study was 27.1%, higher than that in the 5-FU group, which is consistent with previous studies (14).

In contrast to studies included European and American patients, in which angina has been the most common evidence of FIC (23), the most common manifestation in our study was arrhythmia. The incidence rate of arrhythmia was as high as 42% in the 5-FU group, and 50% in the capecitabine group. A previous Chinese study also found the incidence rate of arrhythmia was as high as 20.9% (14). We also found that ST-T changes accounted for a relatively high proportion of FIC. Such electrocardiographic changes could be a potential harbinger for asymptomatic myocardial ischemia or arrhythmia (26). However, because most related heart ischemia resolved in a short time, the incidence rate of FIC might have been higher if continuous electrocardiographic monitoring had been carried out on patients who were treated with fluoropyrimidine.

The incidence of cardiotoxicity had been reported to increase with increasing cycles of chemotherapy (14). But our study found that cardiotoxicity in 5-FU regimen occurred most frequently in the early duration of chemotherapy, consistent with results that FIC was found to occur mostly during the first cycle of chemotherapy (11, 16) suggesting 5-Fu induced FIC may not be the dose-limiting adverse event. In addition, the incidence rate of FIC was higher in patients who were concurrently treated with targeted drugs (bevacizumab, cetuximab). Previous studies also reported that the regimens containing bevacizumab and panitumumab appeared to have a higher cardiotoxicity risk (27). Bevacizumab significantly reduces cardiomyocyte viability and increases cell apoptosis, which in turn increase the incidence rate of the relevant cardiotoxicity (28).

Whether age is associated with FIC was controversial in published studies (14, 24, 29, 30). Our study found the incidence rate of 5-FU induced FIC rose in patients with advanced age (≥60 years). Furthermore, high BMI (>22.97) was correlated with the occurrence of 5-FU induced FIC, which has not been previously reported. Our results might suggest that when using 5-FU-based chemotherapy in patients with high BMI, we should increase the frequency of cardiovascular-related tests.

Previous studies reported that elevation of inflammatory cell such as neutrophils and eosinophilia, as well as inflammation-related indices may contribute to varied cardiovascular disease (31–35). Therefore, we also collected inflammation-related biomarkers (leukocytes, neutrophils, eosinophils, monocytes and Systemic immune immune-inflammation index(SII)) and included them into analysis. We did not find significant correlation between inflammation-related biomarkers and 5-Fu induced FIC. While in patients using the capecitabine-based chemotherapy, elevated neutrophils and eosinophils were found to be independent risk factors for capecitabine induced FIC, which may indicate the occurrence of capecitabine induced FIC is related to pre-existing inflammation.

Nomograms have been used extensively in oncology and medicine to predict the probability of clinical events. They can assist clinicians to make data-based and easily interpreted decisions because of improved accuracy and readily comprehensible predictions (36). The nomogram predicting 5-Fu induced FIC was established based on the four risk factors identified through Lasso regression and the multivariate logistic regression analysis. The area under the ROC curve is 0.751, showing good predictive accuracy and the calibration curve for predicting the occurrence of the FIC in the CRC patients was identical with the actual curve. According to the result of the Hosmer-Lemeshow goodness of fit, The C-index of 0.751 through the internal validation means the nomogram had a good imitative effect and a good degree of discrimination and accuracy. To the best of our knowledge, this is the first study for construct a nomogram for predicting the risk of 5-Fu related FIC in CRC patients.

However, we did not construct nomogram model for predicting capecitabine induced FIC, because of the relatively small number of patients in the capecitabine group, and only two independent high-risk factors identified.

Nonetheless, our study has some limitations. First, although we collected past history on patients with FIC, duration of comorbidities, was not captured and is, therefore, not included in the assessment. Second, this model was developed using retrospective data at a single center, which inevitably suffered from confounding bias. Third, external validation was not undertaken but would certainly be valuable to confirm the performance of the nomogram. However, because the data are collected as a part of routine clinical records, other hospitals that collect similar data could likely apply this model.

## Conclusions

In conclusion, our study demonstrated increased risk of 5-Fu related in early duration of chemotherapy, and in patients with age≥60, high BMI, or treatment with targeted drugs. For patients treated with capecitabine-based regimen, FIC appeared to be related to inflammatory indices. The constructed nomogram showed a high degree of accuracy for prediction of FIC in CRC patients and may eventually help clinicians for risk evaluation.

## Data availability statement

The original contributions presented in the study are included in the article/**Supplementary Material**. Further inquiries can be directed to the corresponding author.

## Ethics statement

The studies involving human participants were reviewed and approved by the Affiliated Cancer Hospital of Guizhou Medical University. The institutional ethics committee waived the requirement for informed consent given the deidentified data.

## Author contributions

YW and WWC designed the study. YW, WWC, WLW, HMD, and WHC contributed to the collection and analysis of the study data. YW wrote the article. HMD, GW, JC and WLW revised the manuscript. All authors contributed to the article and approved the submitted version.
