# Supplementary figures and images for: Risk factors for fluoropyrimidine-induced cardiotoxicity in colorectal cancer: A retrospective cohort study and establishment of a prediction nomogram for 5-FU induced cardiotoxicity
Source: Front Oncol. 2023 Mar 1;13:1017237. doi: 10.3389/fonc.2023.1017237 (PMC10016093; doi:10.3389/fonc.2023.1017237)

Appendix figure 1

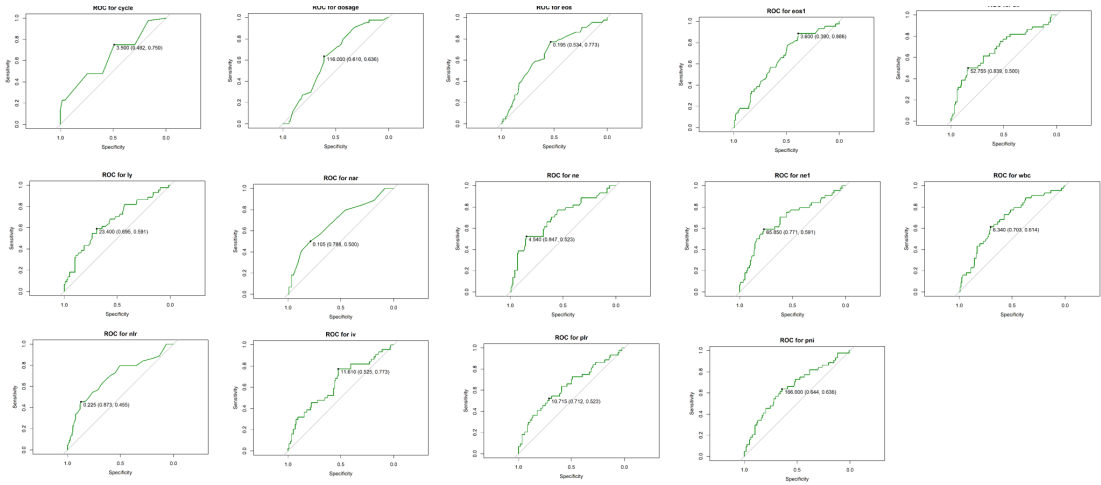

ROC results of capecitabine group

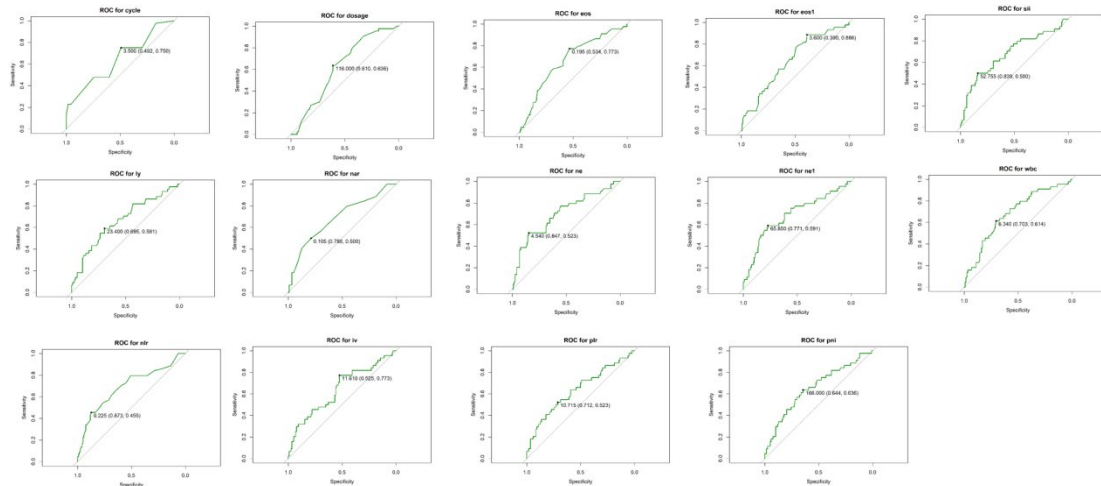

ROC results of 5-FU group

Supplement: Supplementary file 1 [file Image_1.pdf]
